# Aging-Induced Physicochemical Changes in Petroleum- and Biobased Microplastics Influence Depolymerization and Gut Microbiota in *Tenebrio molitor* Larvae

**DOI:** 10.3390/microorganisms14081700

**Published:** 2026-08-03

**Authors:** Yuan Tian, Meng-Qi Ding, Jie Ding, Xin-Ran Ren, Sheng-Qiang Fan, Bing-Feng Liu, De-Feng Xing, Lei Zhao, Zhi-Rong Zhang, Lu-Yan Zhang, Nan-Qi Ren, Shan-Shan Yang

**Affiliations:** 1 National Engineering Research Center for Safe Disposal and Resources Recovery of Sludge, Harbin Institute of Technology, Harbin 150090, China; raintian1990@foxmail.com (Y.T.); dingjie123@hit.edu.cn (J.D.); fsq@hit.edu.cn (S.-Q.F.); lbf@hit.edu.cn (B.-F.L.); dxing@aliyun.com (D.-F.X.); lzhao@hit.edu.cn (L.Z.); rnq@hit.edu.cn (N.-Q.R.); 2 State Key Laboratory of Urban-rural Water Resource and Environment, Harbin Institute of Technology, Harbin 150090, China; 21S029004@stu.hit.edu.cn; 3 State Key Laboratory of Water Engineering Ecology and Environment in Arid Area, College of Water Conservancy and Civil Engineering, Inner Mongolia Agricultural University, Hohhot 010018, China; 4 College of Forestry, Inner Mongolia Agricultural University, Hohhot 010018, China; zhang_zr@126.com; 5 School of Environmental Science and Engineering, Yancheng Institute of Technology, Yancheng 224051, China; zly3161@163.com

**Keywords:** *Tenebrio molitor*, polyethylene, polylactic acid, aging, *Spiroplasma*, *Lactobacillus*

## Abstract

In this study, we evaluated the influence of physicochemical aging on the biological processing and depolymerization performance of polyethylene (PE) and polylactic acid (PLA) by *Tenebrio molitor* larvae, with the goal of improving insect-based plastic treatment strategies. PE and PLA subjected to a sequential freezing–ultraviolet aging protocol showed modest increases in total larval consumption (approximately 11% for PE and 10% for PLA) compared with pristine materials. Aging also accelerated the processes related to chemical depolymerization, as evidenced by Fourier transform infrared spectroscopy and scanning electron microscopy showing the formation of oxidized functional groups and surface structural deterioration, respectively. Gel permeation chromatography indicated significant reductions in molecular weight. In addition, thermogravimetric analysis was used to evaluate the changes in thermal stability associated with polymer degradation. Gut microbiome analysis revealed that plastic diets and aging collectively shaped microbial structure and compositional shifts, with deterministic ecological processes dominating community assembly. PE diets enriched Proteobacteria, while PLA diets enriched Firmicutes and Desulfobacterota. Notably, aging strengthened microbial cooperation and enriched key genera, such as *Spiroplasma* sp. and *Lactobacillus* sp., which are potentially associated with plastic-associated metabolic adaptation. Overall, aging modestly facilitated larval processing and partial depolymerization of both fossil-based and bio-based plastics, as reflected by increased plastic consumption, polymer chain scission, and surface oxidation. It also enhanced the functional robustness of the larval gut microbiome. These findings provide mechanistic insights into insect-mediated plastic processing systems, offering mechanistic guidance for future, combined plastic treatment strategies rather than an immediately scalable stand-alone solution.

## 1. Introduction

Plastic waste has emerged as a critical concern in global environmental and waste management efforts [1]. In 2023, annual plastic production exceeded 400.3 million mt. The six most commonly used petroleum-based plastics include polyethylene (PE), polypropylene (PP), polyvinyl chloride, polyurethane, polyethylene terephthalate, and polystyrene (PS) [2]. The persistent accumulation of these plastics poses significant challenges to ecosystems and human health. Compared to these persistent materials, biodegradable plastics, such as polylactic acid (PLA), polybutylene succinate (PBS), and poly(butylene adipate-co-terephthalate) (PBAT), are generally more susceptible to hydrolytic degradation due to their chemical structures [3]. Currently, PLA accounts for 24% of the global biodegradable plastics market [4], making both PE and PLA widely used polymers and major contributors to global environmental pollution, with high environmental relevance [2,3,5].

Improper plastic waste disposal and environmental weathering processes lead to the formation of microplastics (MPs), with particle sizes less than 5 mm [1,6,7]. These MPs are ubiquitous environmental contaminants and can interact with co-existing pollutants, thereby influencing their environmental fate [8,9,10,11,12,13]. Additionally, MPs undergo secondary weathering induced by environmental factors, such as UV radiation, thermal fluctuations, mechanical stress, and chemical oxidation [1,14,15]. This process generates nanoplastics (NPs) with a smaller particle size and increased surface area, thereby altering physicochemical characteristics, such as functional groups and molecular weight (MW) [16]. These changes may influence the interactions between MPs and microorganisms, potentially affecting subsequent biological processing. For example, PE and PS biodegradation rates increased by 7.8% and 5.1%, respectively, after 45 days of UV treatment, suggesting that photodegradation may enhance the biodegradation of these MPs indirectly by altering their susceptibility to microbial processing, rather than by directly increasing their biodegradation rates [11,17].

Although research on plastic biodegradation has spanned over 70 years [7,18], the microbial transformation of plastics in natural environments remains slow, with degradation often occurring over extended time scales [16]. Aging processes, such as UV exposure, can reduce polymer resistance to biological processing by inducing chain scission and surface oxidation, thereby increasing their susceptibility to microbial attack [19]. Environmental factors, such as freeze–thaw cycles and thermal treatments, also modify the surface and structural properties of plastics, potentially influencing microbial colonization and enzymatic activity [7,19,20,21]. These aging-induced modifications may contribute to reduced persistence of plastics, although their effects on biodegradation remain context-dependent.

In recent years, mealworms (*Tenebrio molitor* larvae) have attracted increasing attention as a model system for investigating insect-mediated plastic processing, with processing efficiency closely linked to the physicochemical properties of target polymers [6,16,22,23]. Studies have shown that the PE and PS biodegradation efficiencies of mealworms are influenced by factors such as the MW, hydrophobicity, and crystallinity of polymers [6,22,23,24]. When a polymer’s MW is high, larvae may show limited plastic processing efficiency, which has been associated with changes in the metabolic activity and structure of the gut microbiome [23]. Moreover, PS aging has been reported to alter larval processing efficiency and gut microbial diversity, suggesting that polymer physicochemical modifications may influence host–microbiome interactions [7]. Overall, environmental aging significantly alters the physicochemical characteristics of MPs [1,8,11,25]. Environmental aging processes, such as sunlight exposure and temperature fluctuations, can reduce the particle size of MPs, thereby influencing their interactions with microorganisms and enzymes [7,18,26,27,28,29,30,31]. However, the underlying biological responses involved in these processes remain incompletely understood. Although natural environmental weathering involves complex and heterogeneous conditions, including full-spectrum solar radiation and seasonal temperature fluctuations, previous studies have primarily focused on either sunlight exposure or insect-mediated degradation. Research on the combined effects of plastic type (fossil-based vs. bio-based) and aging on insect-mediated degradation and gut microbiota dynamic remains limited. This knowledge gap is especially relevant in environments characterized by temperature fluctuations, intermittent freezing, snow cover, variable moisture, and UV exposure. Therefore, further investigation is needed to explore the effects of sunlight exposure and temperature variations on the physicochemical characteristics of different types of MPs and their implications for insect-mediated degradation in high-latitude environments.

In this study, we explore how a sequential freezing–UV aging protocol alters the physicochemical characteristics of PE and PLA and affects mealworm-mediated processing and depolymerization of these plastics. This protocol represents an accelerated laboratory-based aging pre-treatment designed to induce controlled photochemical and freeze–thaw effects under defined conditions, rather than a complete simulation of natural weathering. Our research focuses on aging-induced changes in plastic physicochemical characteristics, gut microbiota assembly patterns, and the influence of plastic type and aging on the responses of the gut microbial community in T. molitor larvae. Our findings provide insights into the interactions between aged MPs and insect-associated microbial systems under controlled laboratory conditions, contributing to the mechanistic understanding of insect-mediated plastic processing.

## 2. Materials and Methods

### 2.1. Source of Mealworms and MPs

The larvae of *T. molitor* used in this study were sourced from Yantai, Shandong Province, China, and were maintained on a wheat bran (WB) diet. The larvae measured from 1.5 to 2.0 cm in length, with an average weight of 61.6 ± 0.9 mg (Figure S1). PE and PLA were sourced from Dongguan, Guangdong Province, China. PE had a density of 0.95 g/cm^3^ and a particle size below 48 μm, while PLA had a density of 1.25 g/cm^3^ and a particle size below 150 μm. These MPs were used without additional sterilization treatment prior to feeding experiments. Plasticizers in both PE and PLA were under the detection limit of <0.05 ppm (*w*/*w*) [5,32]. The number-average MW (M_n_) of PE was 8.6 kDa, with a weight-average MW (M_w_) of 124.4 kDa. Similarly, PLA exhibited an M_n_ of 132.7 kDa and an M_w_ of 186.6 kDa (Table S1).

### 2.2. PE and PLA Depolymerization Experimental Design

In this study, we investigated the effects of sequential freezing–UV pre-treatment on the physicochemical characteristics of PE and PLA and how these changes influenced subsequent larva-mediated transformation and depolymerization. Our procedure followed the same aging protocol used in our previous research on PS degradation [7], which employed environmentally relevant temperature ranges derived from field conditions in Harbin. In this region, the average winter temperature is −20 ± 2 °C, with approximately 10 h of daylight in January, while the average summer temperature (July) is 25 ± 2 °C. UV_254_ irradiation does not fully reproduce the spectrum and intensity of natural sunlight. However, in this study, it was used as a laboratory proxy for the high-energy UV fraction responsible for polymer chain scission. The aging protocol consisted of repeated UV–freeze cycles. Each cycle involved 10 h of UV_254_ irradiation using four 6 W UV lamps (UV-Tec, Philips, Shanghai, China), with a peak emission wavelength of 230 nm. The lamps were positioned 10 cm above the samples in a dark chamber, followed by freezing at −20 °C for 14 h. This UV–freeze cycle was selected to maintain consistency with our previous study on PS aging [7]. The cycle was repeated 10 times to accelerate physicochemical aging processes, including surface oxidation and thermally induced structural rearrangements. Because natural aging also involves rainfall, snow cover, humidity, soil contact, mechanical abrasion, and fluctuating radiation intensity, the present protocol should be interpreted as a controlled model for isolating UV/freezing effects rather than as a full environmental aging reproduction. PE and PLA samples (60 g each) were evenly distributed in Petri dishes (15 cm diameter) during treatment. The experimental groups included pristine PE (PEcon), pre-treated PE (PE1), pristine PLA (PLAcon), and pre-treated PLA (PLA1). The protocol is summarized in the graphical abstract. This design improves experimental comparability with the previous PS aging system while limiting the interpretation to accelerated photochemical and freeze–thaw effects under controlled laboratory conditions.

Our study focused on simplified dietary conditions to isolate the effects of plastic type and aging treatment on plastic processing by *T. molitor* and its gut microbiota, rather than optimizing co-feeding-based depolymerization efficiency. To assess the effects of pre-aging on the depolymerization of PE and PLA, as well as on the gut microbiota of *T. molitor*, larvae were assigned to five feeding treatments: PEcon, PE1, PLAcon, PLA1, and WB. Approximately 150 larvae were randomly selected and introduced into food-grade PP containers (10.3 × 6.5 × 6.5 cm) at an initial density of ~2 larvae cm^−2^. Feed substrates were prepared by homogenizing 12.5 g of PE/PLA powder with agar (55 mL, 3%, *w*/*w*) following the previously reported method [7,22]. Agar was used solely as an inert gelling/structural matrix to immobilize plastic particles and ensure uniform ingestion. All experimental groups, including the WB control, contained identical concentrations of agar to eliminate nutritional bias introduced by the matrix. Dead larvae were promptly removed to reduce cannibalism [5]. All groups were maintained in an artificial climate chamber (250 L) for 24 days at a temperature of 27 ± 0.5 °C and a relative humidity of 70 ± 5% under dark conditions. The feeding duration of 24 days was selected to maintain consistency with our previous study on PS aging and mealworm-mediated depolymerization, enabling direct comparability of the degradation performance and microbiome responses under standardized experimental timelines. Each container initially received 10 g of diet, and ad libitum access to feed was ensured throughout the experiment. All treatments were conducted in triplicate.

Measured parameters included larval survival rate (SR), average larval weight, total PE/PLA consumption, and specific plastic consumption rate (SPCR) (Supplementary Materials Method S1). Frass was also collected for further analysis. The surface morphology of frass and residual plastic was characterized using scanning electron microscopy (SEM; ZEISS Sigma 500, Carl Zeiss Microscopy GmbH, Jena, Germany) (Supplementary Materials Method S2).

### 2.3. Characterization of PE and PLA Depolymerization by Larvae

Pristine, aged, and residual polymer samples were extracted and analyzed to characterize the depolymerization of PE and PLA. For residual polymers recovered from frass, polymer extraction was performed before instrumental analysis to separate the target polymer fraction from insoluble organic residues, including microbial biomass and diet-derived material, as far as possible. For PE, 1,2,4-trichlorobenzene was used as the extraction solvent, and the recovered polymer phase was separated, washed, dried, and then used for FTIR and high-temperature gel permeation chromatography (HT-GPC; PL GPC 220, Agilent Technologies, Santa Clara, CA, USA). For PLA, samples were extracted using chloroform, and the recovered polymer phase was separated, washed, dried, and then used for FTIR and GPC (PL GPC 50, Agilent Technologies, Santa Clara, CA, USA).

Fourier transform infrared spectroscopy (FTIR; Nicolet iS50, Thermo Fisher Scientific, Waltham, MA, USA) was performed to identify chemical modifications, particularly the formation of oxidative functional groups, as well as the structural changes in residual PE and PLA. Thermogravimetric analysis (TGA; NETZSCH STA 449 F3, NETZSCH-Gerätebau GmbH, Selb, Germany) was conducted to evaluate the thermal stability of polymer powder samples heated from 40 to 800 °C at 10 K min^−1^ under a high-purity nitrogen atmosphere (99.999%). Water contact angle (WCA) was measured for pristine and aged polymers using a Dataphysics DCAT21 instrument (Heilna Trade GmbH, Neu-Isenburg, Germany). These established procedures were applied to assess plastic biodegradation [16,29]. All analyses were performed in triplicate. Additional information is provided in Supplementary Materials Method S3–S5.

### 2.4. Gut Microbiome Analysis

At the end of the 24-day experiment, 30 larvae were randomly collected from each treatment group for gut microbial community analysis. Prior to gut dissection, larvae were starved for 24 h to allow for the clearance of transient gut contents and reduce contamination from residual diet particles. To avoid external contamination, the larvae were rinsed with 75% ethanol and then a sterile PBS (Phosphate-Buffered Saline) solution for surface sterilization. Dissections were conducted in a laminar flow biosafety cabinet using sterilized instruments to minimize environmental contamination (detailed in Supplementary Materials Method S6). Gut microbial DNA samples were sequenced on the Illumina Hiseq 6000 platform. Sequence data were processed and analyzed using the Majorbio Cloud Platform (https://www.majorbio.com, accessed on 1 October 2025).

### 2.5. Statistical Analysis

The statistical analyses were performed using Origin (version 2024). Data are presented as mean ± standard deviation (SD). One-way analysis of variance (ANOVA) was used to assess differences in SR, average larval weight, total PE/PLA consumption, SPCR, and the changes in Mn and Mw among diet groups. When significant effects were detected, post hoc pairwise comparisons were conducted using Fisher’s least significant difference (LSD) test for hypothesis-driven comparisons among predefined treatments [33]. Because LSD is relatively liberal and does not control the false discovery rate across all possible comparisons, statistically significant results were interpreted together with effect size, consistency among independent indicators, and biological plausibility. Differences in microbial community structures were analyzed using distance-based redundancy analysis (db-RDA) and permutational multivariate analysis of variance (PERMANOVA) based on relative abundance data and Bray–Curtis distance matrices with 999 permutations. Variance partitioning analysis (VPA) was performed to evaluate the relative contributions of explanatory variables to community variations. Network analyses were conducted using Spearman correlation-based methods (correlation cutoff: 0.5). The topology parameters of the network were used to identify key taxa (module hubs and connectors).

## 3. Results and Discussion

### 3.1. Effects of Aging Treatment on the Physicochemical Characteristics of PE and PLA

**Figure 1:**
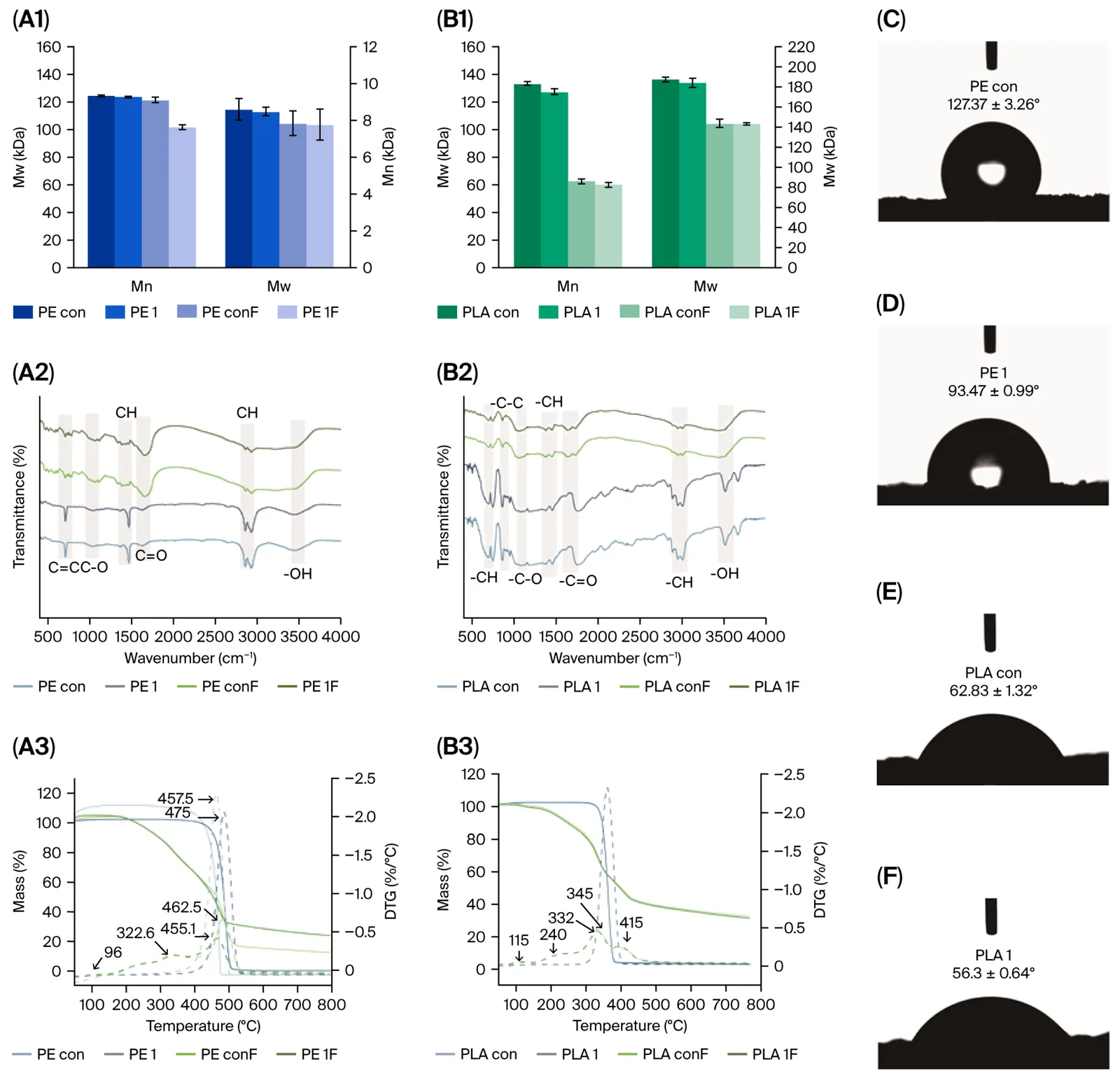
Effects of aging treatment on PE and PLA: (**A1**,**B1**) GPC of PEcon, PLAcon, PE1, PLA1, and the residual polymers from larval frass fed with plastic (frass = F); (**A2**,**B2**) FTIR spectra of PEcon, PLAcon, PE1, PLA1, and the residual polymers from larval frass fed with plastic; (**A3**,**B3**) TGA of PEcon, PLAcon, PE1, PLA1, and the residual polymers from larval frass fed with plastic; (**C**–**F**) WCA of PEcon, PLAcon, PE1, and PLA1.

In this study, UV irradiation was applied as an accelerated proxy for approximately 30 days of winter sunlight exposure in Harbin (details in Supplementary Materials Method S7). After freezing–UV-induced aging, both PE and PLA exhibited a light-yellow coloration, deviating from the white appearance of their pristine counterparts (Figure S1). SEM observations revealed visible surface defects in both polymers, including irregular structures, pits, and localized surface deterioration (Figure S1). GPC indicated that the Mn and Mw of PE1 were lower compared to pristine PEcon (Figure 1(A1)), suggesting that aging induced the depolymerization of PE macromolecules. A similar trend was observed for PLA, with the Mn and Mw of PLA1 being lower than those of PLAcon (Figure 1(B1)). These observations are consistent with the findings of previous studies on PS aging [7]. Aging also affected the surface hydrophobicity of polymers, as evidenced by the changes in their WCAs (Figure 1C–F). Hydrophobic surfaces hinder enzyme adsorption and catalytic activity, which are essential for polymer depolymerization [6,7,22]. Specifically, the WCA of PEcon was 127.37 ± 3.26° (n = 3), whereas PE1 exhibited a 26.62% reduction in WCA (Figure 1C,D). Similarly, aging reduced the WCA of PLAcon by 10.39% (Figure 1E,F). The more significant change in the hydrophobicity of PE may be related to its smaller particle size, highlighting the potential influence of particle size on the bio-transformation and depolymerization behavior of MPs.

FTIR spectra and TGA provided further insights into the effects of aging treatment. In the FTIR spectrum of PE, the peak at 717 cm^−1^ displayed fluctuations, indicating the disruption of the C=C bond (Figure 1(A2)) [5]. In aged PLA, the absorption peaks at 1708 cm^−1^ and 3398 cm^−1^ corresponding to carbonyl and −OH groups, respectively, intensified and broadened, reflecting changes in these functional groups (Figure 1(B2)) [3]. TGA revealed that PEcon exhibited mass loss within the temperature range of 362.5–492.5 °C, whereas the corresponding range for PE1 shifted to 382.5–517.5 °C (Figure 1(A3)). PLA displayed a largely consistent thermal behavior before and after aging, although PLA1 exhibited a slightly higher mass loss compared to PLAcon, reaching 96.5% (Figure 1(B3)). Collectively, these results indicate that aging treatment altered the thermal stability of PE and PLA to varying degrees, potentially enhancing depolymerization by promoting surface cracks and functional group transformations. In particular, UV-induced surface damage and reduced WCA likely facilitated microbial and enzymatic attachment, accelerating polymer depolymerization. It should be noted that the freezing–UV aging protocol used in this study represents an accelerated laboratory weathering model rather than a direct simulation of natural environmental conditions. Although UV_254_ does not fully replicate the solar spectrum, this approach effectively induces key physicochemical changes, such as oxidation and surface fragmentation, which are commonly observed in naturally weathered plastics [34]. Accordingly, the observed physicochemical shifts should be viewed as indicators of accelerated weathering potential rather than quantitative predictions of degradation under field conditions.

### 3.2. Larval Consumption and Depolymerization of PE and PLA After Aging Treatment

**Figure 2:**
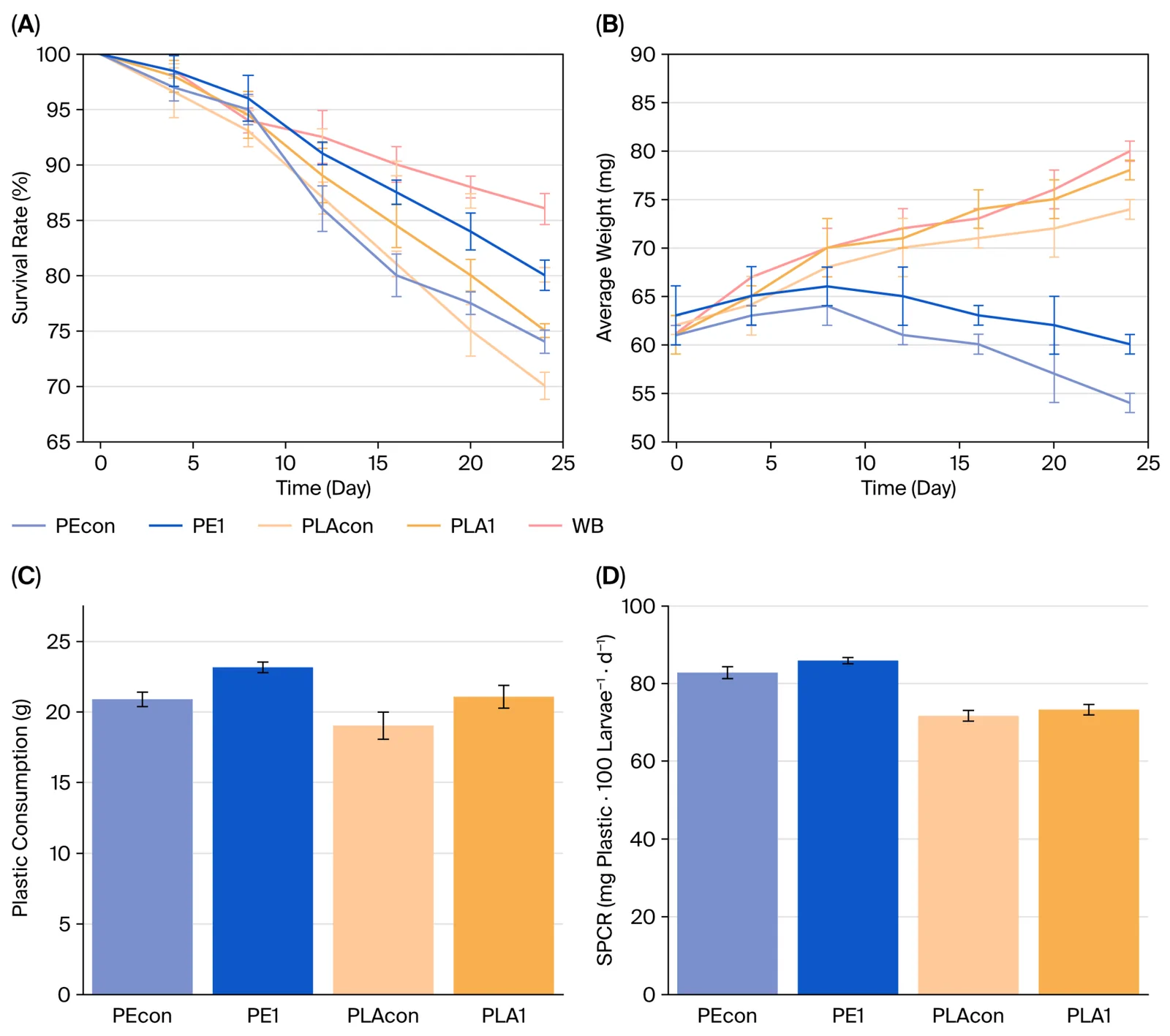
Consumption and depolymerization of PE and PLA by *T. molitor* larvae: survival rates (**A**) and average weight change (**B**) for *T. molitor* larvae in PEcon, PLAcon, PE1, PLA1, and WB groups; comparison of plastic consumption (**C**) and specific plastic consumption of 100 larvae every day (SPCR, mg plastic·100 larvae^−1^·d^−1^) (**D**) in PEcon, PLAcon, PE1, and PLA1 groups (all values represent mean ± SD, n = 3).

To evaluate the biological processing and degradation potential of aged PE and PLA, bioassays were conducted using *T. molitor* larvae. Mealworms were fed WB, pristine MPs, and aged MPs over 24 days (Figure S1). Aging increased the apparent depolymerization-related responses of both MPs compared to their pristine counterparts, consistent with the structural alterations that may influence the depolymerization performance of larvae [6,7,22]. Throughout the experiment, larvae actively consumed both PE and PLA. After 24 days, the SR of PEcon-fed larvae (74 ± 1.0%) was lower than both the WB control (86 ± 1.4%) and the aged PE1 group (80.0 ± 1.4%), indicating ingestion-based utilization of PE. A similar trend was observed for PLA, with PLAcon and PLA1 showing SR values of 70 ± 1.0% vs. 75 ± 0.64% (Figure 2A and Table S1).

PE and PLA consumption by larvae increased progressively during the 24-day period. In the PEcon and PE1 groups, larvae consumed 20.9 ± 0.5 g and 23.2 ± 0.4 g of PE and PLA, respectively, while in the PLAcon and PLA1 groups they consumed 19.1 ± 0.9 g and 21.1 ± 0.8 g of the respective polymers (Figure 2C and Table S2). Consistent with these trends, the plastic removal rate was slightly higher in the aged groups. The PE1 group showed a slightly higher removal rate than PEcon (55.6 ± 5.0% vs. 53.8 ± 2.4%), while PLA1 exhibited a modest improvement over PLAcon (81.6 ± 0.4% vs. 80.8 ± 3.9%) (Table S2). The SPCR of larvae in the PE1 group (859.3 ± 7.6 mg PE 100 larvae^−1^·d^−1^) was approximately 4% higher than that in the PEcon group, while the SPCR in the PLA1 group (732.8 ± 12.2 mg PLA 100 larvae^−1^·d^−1^) was 2% higher than that in the PLAcon group (Figure 2D and Table S2). These results indicate that aged plastics were slightly more readily processed by the larvae, likely due to freezing–UV-induced physical and chemical alterations, such as cracking and the exposure of oxygen-containing functional groups, which may enhance polymer accessibility. However, the magnitude of the improvement was modest, indicating that aging alone is unlikely to be sufficient for practical plastic treatment and may need to be combined with nutritional co-feeding, microbial enrichment, or other process-optimization strategies [7]. Relative to earlier reports showing UV-enhanced PE and PS biodegradation or co-diet-assisted PE transformation [7], the improvement produced by freezing–UV aging in this study was measurable but limited in terms of the consumption and removal rate.

During the 24-day experiment period, larvae in the WB, PLAcon, and PLA1 diet groups displayed weight gains of 31.1 ± 1.1%, 27.9 ± 1.0%, and 19.35 ± 1.3%, respectively, whereas larvae in the PEcon and PE1 groups experienced weight losses of 11.5 ± 0.1% and 4.8 ± 1.2% (*p* < 0.05), respectively (Figure 2B and Table S2). These results indicate that PE alone could not provide sufficient nutritional value to sustain normal larval growth development, while PLA could support larval development, aligning with previous studies on PLA biodegradation by mealworms [3,6]. Overall, although *T. molitor* exhibited degradation potential toward both PE and PLA, depolymerization efficiency and larval physiology were strongly influenced by polymer type and aging treatment. Future work should focus on optimizing larval bio-transformation performance while maintaining insect health, thereby contributing to the development of biologically assisted strategies for plastic waste management.

Compared with our previous study on PS degradation by *T. molitor* larvae [7], the enhancement effect induced by freezing–UV aging observed in the present study was relatively comparable but polymer-dependent. In the previous PS system, UV–freezing aging increased specific PS consumption from 563.9 to 602.8 mg PS·100 larvae^−1^·d^−1^ and increased PS removal efficiency from 48.7% to 49.6%, corresponding to an improvement of approximately 6.9%. In the present study, aging increased PE and PLA consumption by approximately 11% and 10%, respectively, while the improvement in SPCR was approximately 4% for PE and 2% for PLA. These results indicate that, while aging pre-treatment generally enhances insect-mediated plastic processing by reducing polymer resistance through chain scission, oxidation, and surface modification, the magnitude of improvement depends strongly on polymer properties, including chemical structure, hydrophobicity, and susceptibility to depolymerization. Compared with PS, PE exhibited a stronger response to aging-induced improvement, whereas PLA showed limited enhancement due to its inherently higher degradability.

### 3.3. Depolymerization and Oxidation of PE and PLA by Mealworms

SEM, GPC, FTIR, and TGA analyses were used to evaluate the larval-mediated biological transformation, depolymerization, and oxidation of PE and PLA [16]. Mechanistically, PE transformation is expected to begin with aging-induced oxidation and chain scission at the polymer surface, which reduce hydrophobicity and increase defect sites accessible to gut-associated microbes and enzymes. PLA transformation is more strongly associated with ester-bond cleavage and hydrolysis, producing lower-molecular-weight fragments that can be further metabolized by the gut microbiota. SEM imaging revealed morphological deterioration in both polymers after passing through the larval gut, with aged MPs exhibiting more visible surface defects, including irregular structures, pits, and localized roughened regions, than pristine MPs (Figure S1). These features suggest an enhanced susceptibility of MPs to microbial and enzymatic attack in the gut, consistent with the reduced hydrophobicity via WCA measurements (Figure 1C–F). These findings collectively indicate that aging altered surface physicochemical characteristics and facilitated biological processing, highlighting the potential of aging treatments to facilitate plastic depolymerization and structural transformation, especially under aging-induced surface oxidation and mechanical damage. Thus, the mechanistic interpretation is polymer-specific: aged PE is mainly sensitized through oxidative surface activation and defect formation, whereas aged PLA is more directly affected by hydrolysable ester-bond cleavage and the generation of lower-molecular-weight fragments.

It should be noted that SEM was used as a qualitative supplementary characterization rather than the sole evidence for polymer degradation [35]. Therefore, surface morphological changes were interpreted together with GPC, FTIR, TGA, and WCA results, which collectively support the physicochemical alteration and partial depolymerization of PE and PLA after aging and larval processing. More detailed microscopic analyses, such as quantitative surface morphology measurements and higher-resolution imaging, will be considered in future studies.

GPC analysis confirmed substantial decreases in M_n_ and M_w_ after larval feeding (Figure 1(A1,B1)). PLA showed considerably higher biodegradability than PE after 24 days, consistent with earlier reports [5,6,36]. For PE, M_n_ decreased by 2.62% (PEcon) and 18.36% (PE1), while M_w_ decreased only slightly. In contrast, PLA exhibited pronounced scission in the polymer chain, with M_n_ reductions of 53.18% (PLAcon) and 54.87% (PLA1) and M_w_ reductions of 23.28% (PLAcon) and 23.51% (PLA1). These results indicate that aging likely enhanced the enzymatic accessibility and biological processing susceptibility of both polymers, although the effect was more apparent on PE due to its lower MW and smaller particle size.

FTIR spectra provided further evidence of the oxidation and depolymerization of PE and PLA (Figure 1(A2,B2)). In the FTIR spectrum of PE, the characteristic stretching peaks of C-H at 2921 cm^−1^ and 2852 cm^−1^ diminished markedly, and the peak at 1470 cm^−1^ disappeared in the mealworm frass. New oxygen-containing functional groups—including C=O (~1700 cm^−1^) and -OH (~3300 cm^−1^)—were detected in the extracted residual PE fraction from frass, reflecting oxidation-induced hydrophilization [5] (Figure 1(A2)). Although solvent extraction substantially reduced interference from organic residues, the possible contribution of tightly associated metabolites or microbial-derived products cannot be completely excluded [37]; therefore, these bands were interpreted together with GPC, TGA, and SEM evidence rather than as stand-alone proof of complete polymer mineralization. For this reason, the newly observed C-O- and -OH-related FTIR signals in residual PE were treated as supportive evidence of oxidation-related transformation only when they were consistent with molecular weight reduction, thermal profile changes, and SEM-observed surface deterioration. For PLA, depolymerization resulted in the attenuation of ester-associated peaks (1260, 1127, and 1080 cm^−1^) and carbonyl absorption at 1708 cm^−1^ [38], along with broadening of the -OH band at 3398 cm^−1^, indicating ester-bond cleavage and hydroxyl formation (Figure 1(B2)). Co-feeding of polymers and WB has also been reported to support better digestion and metabolism [5,39]. In this study, the physical breakdown of the aged polymers likely contributed to the increased biological processing rates. These findings highlight the potential of aging treatments to promote polymer chain modification and partial depolymerization occurring during passage through the larval digestive system.

TGA results showed the biodegradation of both PE and PLA in the larval digestive tract (Figure 1(A3,B3)). TGA demonstrated that the PEcon exhibited mass loss exclusively within the temperature range of 345–490 °C, with the maximum decomposition rate observed at 457.5 °C. The frass samples from PEcon- and PE1-fed mealworms exhibited mass losses of 57.62% and 44.30%, respectively, within the specified temperature range. The maximum mass loss occurred at 462.5 °C and 455.1 °C, while the maximum decomposition rate was observed at both 95 °C and 322.6 °C (Figure 1(A3)). In contrast, PLA also exhibited a single weight loss interval in the temperature range of 325–415 °C, with the maximum decomposition rate observed at 365 °C. The frass from PLAcon- and PLA1-fed *T. molitor* exhibited a similar trend of mass loss, with maximum losses at 115, 240, 332, 345, and 415 °C (Figure 1(B3)). The decomposition observed within the temperature range of 100–370 °C was attributed to two processes: residual plastic biodegradation and cannibalism in the digestive tract of *T. molitor* [7,40]. These results indicated the presence of residual polymer components and possible transformation products, suggesting the occurrence of partial polymer breakdown during passage through the larval digestive system. Collectively, these observations suggest that the larval digestive system facilitated polymer depolymerization and oxidation-related transformations, while aging treatments exerted only a limited enhancement effect on these processes. Therefore, aging treatments appear to promote plastic depolymerization and, to some extent, improve biological processing efficiency.

### 3.4. Diversity and Shift in the Gut Microbial Community of T. molitor

To identify microbial taxa potentially associated with the plastic processing of aged PE/PLA and evaluate the responses of gut microbiota to different plastic diets, microbial diversity in the gut contents of larvae in different diet groups was analyzed (Table S3). A total of 604,875 sequences were obtained across all five groups, with a coverage rate of 99%. The Simpson and Shannon indices indicated an increase in gut microbial diversity following ingestion of the four plastic diets (Table S3). The Ace, Chao, and Sobs indices indicated a decline in microbial species richness in the PEcon and PE1 groups, while an increase occurred in the PLAcon and PLA1 groups (Table S3). This suggests that the microbial communities in the PEcon and PE1 groups were dominated by a few taxa, whereas the PLAcon and PLA1 groups exhibited a more even distribution of genera, reflecting distinct adaptive responses of the gut microbiota to different plastic diets.

**Figure 3:**
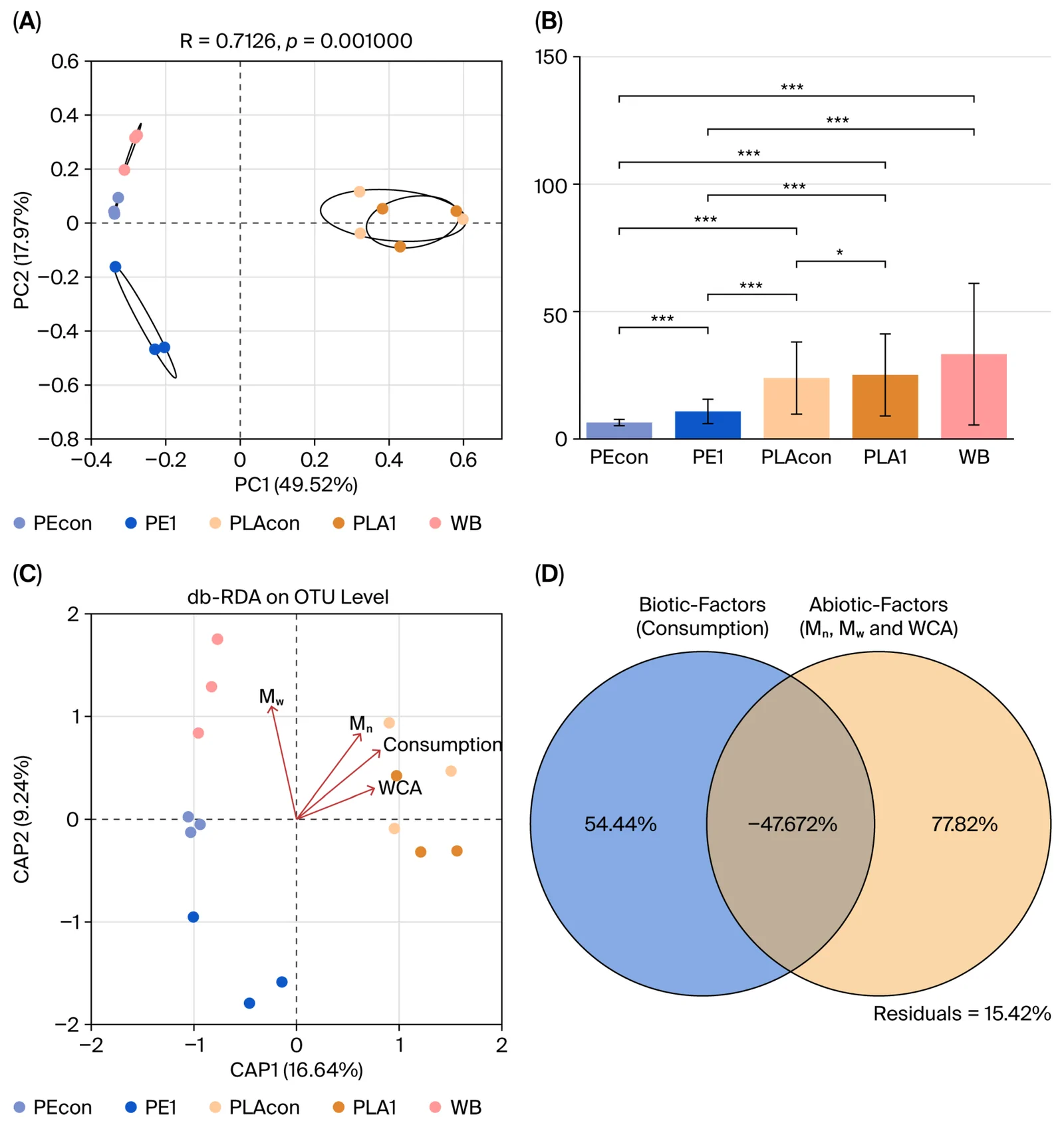
Shift in gut microbial structure due to different diet conditions: (**A**) results of PCA of gut microbial communities in PEcon, PLAcon, PE1, PLA1, and WB groups; (**B**) microbial community assembly or community structure in different groups, as revealed by Normalized Stochasticity Ratio (NST) model (* = *p* < 0.05 and *** = *p* < 0.001); (**C**) distance-based redundancy analysis (db-RDA) showing the effect of M_n_, M_w_, WCA, and larval consumption on the microbial community composition in various groups. The total variance (in percent) explained by each axis is indicated in parentheses. The arrows significantly explain the observed compositional variation in the gut microbial community (PERMANOVA for RDA) across treatments; (**D**) results of VPA showing the contributions of abiotic factors (M_n_, M_w_, and WCA) and biotic factors (larval consumption) to microbial community structures in the gut of *T. molitor* larvae. Samples were obtained on day 24 at the end of the test.

Principal coordinate analysis (PCoA) based on operational taxonomic unit (OTU) levels using Bray–Curtis dissimilarity revealed the clustering patterns associated with different diets (Figure 3A). The gut microbial communities of larvae in the PEcon, PE1, and WB groups were clustered separately, indicating that PE-consuming larvae had a unique gut microbial composition influenced by the aging treatment of the plastic. This finding is consistent with the significant changes in the physicochemical characteristics of pristine and aged PE (Figure 1). In contrast, PLAcon and PLA1 groups were clustered closely together and separately from PEcon, PE1, and WB groups, suggesting that the relatively minor changes in the physicochemical characteristics of pristine and aged PLA had little effect on the microbial community. The energy and nutrient availability in the PE, PLA, and WB diets exerted a significant influence on the abundance and ecological interactions of microbial genera in the gut of *T. molitor*, aligning with previous research [1,5,7,22,36,39]. These findings suggest that the variations in gut microbial communities among larvae of different groups were influenced by their distinct dietary compositions, which subsequently influenced gut microbial interactions, resulting in distinctive metabolic effects. These effects helped alleviate the nutritional deficiency associated with diets consisting solely of plastic.

### 3.5. Microbial Community Composition and Sensitivity

The assembly and diversity of microbial communities are influenced by both stochastic and deterministic ecological processes. To fully understand the diversity and functionality of gut microbiomes, both ecological processes should be considered [22,41,42,43,44]. In this study, a robust tool based on a universal null model framework was employed to quantitatively assess the ecological stochasticity of gut microbiota in *T. molitor* larvae across different diet groups [45]. The Normalized Stochasticity Ratio (NST) model was used to distinguish deterministic (<50%) and stochastic (>50%) community assembly processes, with a threshold value of 50% [46]. The results indicated that the NST values were below 0.5 in all five diet groups, suggesting that deterministic ecological processes dominated the assembly of gut microbiota (Figure 3B). This finding is consistent with previous studies, which showed that the assembly of gut microbial communities in insects is primarily driven by host diet and species, rather than by a single dominant process [5,22]. PERMANOVA and VPA based on distance-based redundancy analysis (dbRDA) (Figure 3C,D) were performed to compare the responses of the gut microbiome to cumulative plastic consumption (biotic factors) and the physicochemical characteristics of PE and PLA (e.g., WCA, M_n_, and M_w_—abiotic factors) [5]. These analyses were used to evaluate the sensitivity of the gut microbiota. The results indicated that both the physicochemical characteristics of plastics and cumulative plastic consumption influenced the composition of the gut microbiota. Although plastic additives in both PE and PLA samples were below the detection limit (<0.05 ppm), their potential influence on gut microbial community composition cannot be completely excluded. Trace additives or degradation byproducts released during aging treatment or gut passage likely exerted selective pressure on microbial populations, potentially contributing to the observed shifts in community structure. However, given their extremely low concentration, their contribution is expected to be minor compared to the dominant effects of polymer physicochemical properties and dietary composition. The unexplained portion of the VPA suggests that, beyond biotic and abiotic factors, stochastic processes also influenced community structure to some extent. Future research should explore how stochastic processes interact with other ecological factors to shape the structure of gut microbiomes.

### 3.6. Response of the Core Microbiomes in the Gut of Mealworm to Different Diets

**Figure 4:**
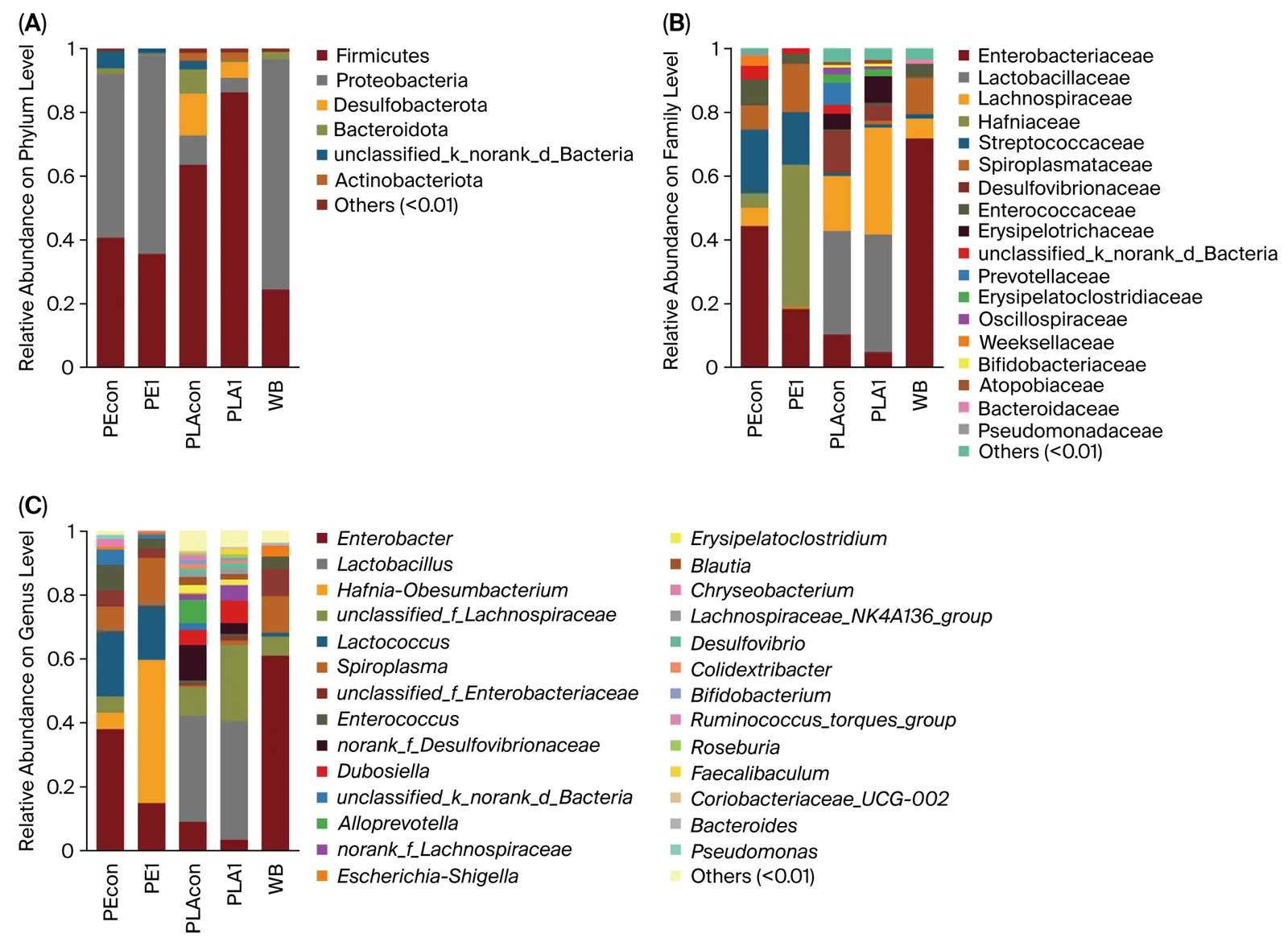
Microbial community composition at the phylum (**A**), family (**B**), and (**C**) genus level in the gut of *T. molitor* larvae. Samples were obtained on day 24 at the end of the test.

The relative abundance analysis visually demonstrated the impact of different diets (such as PE, PLA, and WB) on the gut microbiota of *T. molitor*. Among all treatment groups, Proteobacteria and Firmicutes were the dominant phyla in the gut microbiota of the larvae (Figure 4A), with a combined abundance exceeding 60%. Proteobacteria was significantly enriched in the PE and WB groups, exhibiting dominance, with relative abundances of 72.96% in the WB group, 62.82% in the PE1 group, and 51.18% in the PEcon group. In contrast, the abundance of Proteobacteria dropped significantly to 4.74% in the PLAcon group and 9.91% in the PLA1 group, suggesting that Proteobacteria may contribute to PE degradation. Compared to the WB group (24.10%), Firmicutes was obviously enriched in the plastic-fed groups, with relative abundances of 35.67%, 41.14%, 86.20%, and 63.15% in the PE1, PEcon, PLA1, and PLAcon groups, respectively. This indicated the dominance of Firmicutes in the PLA-based diet groups, suggesting that it may play an important role in PLA depolymerization. Notably, Desulfobacterota was undetected in the PE-fed groups and had a relative abundance of only 0.1% in the WB group. However, it was significantly enriched in the PLA-fed groups, with abundances of 4.77% in the PLA1 group and 12.74% in the PLAcon group. This suggests that Desulfobacterota may be a candidate taxon involved in the depolymerization of PLA and its metabolic byproducts, as it has been reported to encode PHA depolymerase, which is potentially associated with the degradation of bio-plastics [47]. Firmicutes and Bacteroidetes were present across all five dietary treatments, and their ratio (F/B) was closely related to the metabolic potential of the microbiota [7,48]. The F/B ratios were 16.17 and 7.92 in the PEcon and PLAcon groups, respectively. However, in the PE1 and PLA1 groups, the F/B ratio significantly increased, reaching 116.45 and 151.77, respectively (Figure 4A). These findings indicate that changes in the F/B ratio (indicating the potential metabolic capacity of the gut microbiome) also highlight the importance of aging in plastic depolymerization. Physical and chemical changes in aged plastics, such as surface cracks and oxidation, may facilitate microbial colonization or enzymatic reactions, thereby enhancing the metabolic capabilities of the microbial community.

At the family level (Figure 4B), Hafniaceae and Streptococcaceae dominated the gut microbiome in the PE groups, with both taxa demonstrating significant potential for plastic depolymerization [16]. Interestingly, while the relative abundance of Spiroplasmataceae in the PE1 group was significantly higher compared to that in the WB control group, its abundance decreased and was inhibited in the PEcon group. This suggests that the aging of PE may alter the ecological niche of Spiroplasmataceae in the gut, promoting its colonization and potentially enhancing its activity, which may be closely related to the higher plastic depolymerization efficiency of larvae observed in the PE1 group. This phenomenon aligns with the lower species richness in the PE1 group (Table S3). In contrast, different bacterial families were dominant in the PLA diet groups. After 24 days of the experiment, Lactobacillaceae, Lachnospiraceae, and Oscillospiraceae were identified as potential PLA-degrading bacteria [3,49]. Furthermore, *Lachnospiraceae*, *Bifidobacteriaceae*, and *Atopobiaceae*, known as probiotic families regulating gut metabolism, were obviously enriched in the PLA-fed groups [50,51,52,53], particularly in the PLA1 group. This suggests that aged plastic positively influenced the gut microbiota of the larvae. Erysipelotrichaceae and Erysipelatoclostridiaceae, typically considered pathogens in mammalian guts [53], were also enriched in the PLA diet groups in this study, indicating their potential roles in the metabolism of PLA and its depolymerization byproducts. Additionally, *unclassified_k__norank_d__Bacteria* sp. was enriched in the PE and PLA diet groups. The enrichment of this family has also been reported during the biodegradation of PS [7], suggesting that it might be a broad-spectrum candidate taxon.

**Figure 5:**
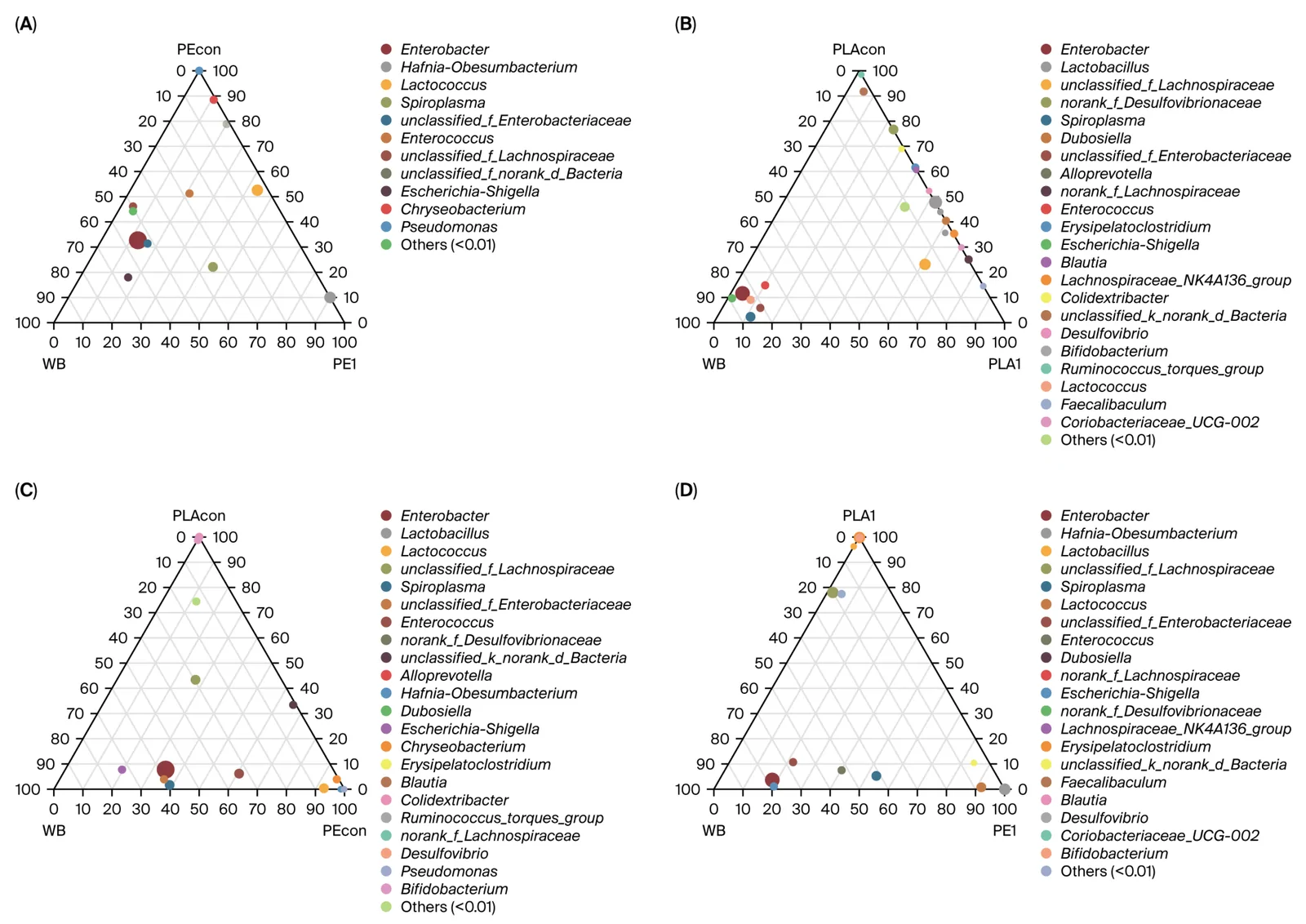
Differential relative abundance of the genus level in the guts of *T. molitor* larvae based on ternary. Samples were obtained on day 24 at the end of the test, the size of the circle represents the average relative abundance of the species. Changes in the composition and distribution of dominant genera among the (**A**) PEcon, PE1, and WB groups; (**B**) PLAcon, PLA1, and WB groups; (**C**) PLAcon, PEcon, and WB groups; (**D**) PLA1, PE1, and WB groups.

At the genus level, the key microbial taxa in the gut of *T. molitor* larvae were significantly different across the experimental groups (Figure 4C and Figure 5). Compared to the WB group, the PE groups showed an enrichment of *Pseudomonas* sp., *Chryseobacterium* sp., *unclassified_k__norank_d__Bacteria* sp., *Hafnia-Obesumbacterium* sp., *Enterococcus* sp., *Lactococcus* sp., and *Spiroplasma* sp., indicating the close associations of these genera with PE depolymerizaton [5,40]. Notably, *Hafnia-Obesumbacterium* sp. and *Spiroplasma* sp. exhibited higher colonization in the guts of larvae in the PE1 group, suggesting that the aging of PE enhanced the ecological niche of these genera, which may be linked to the greater depolymerization efficiency observed in this group (Figure 5A). In the PLA diet group, aging promoted the enrichment of functional genera, such as *Lactobacillus* sp., *unclassified_f__Lachnospiraceae* sp., and *norank_f__Lachnospiraceae* sp., which have been reported to be associated with plastic degradation [3,16,36] (Figure 5B). A comparative analysis revealed that the key genera associated with the PLAcon diet included *Lactobacillus* sp., *unclassified_f__Lachnospiraceae* sp., *norank_f__Desulfovibrionaceae* sp., *Dubosiella* sp., *Erysipelatoclostridium* sp., *Blautia* sp., *Colidextribacter* sp., and *norank_f__Lachnospiraceae* sp. In contrast, the PEcon diet was associated with *Lactococcus* sp., *unclassified_k__norank_d__Bacteria* sp., *Chryseobacterium* sp., *Enterococcus* sp., and *Pseudomonas* sp. After aging, the dominant genera shifted, with the PLA1 group showing enrichment of *unclassified_f__Lachnospiraceae* sp., *Lactobacillus* sp., and *Dubosiella* sp., while the PE1 group exhibited a dominance of *Spiroplasma* sp. and *unclassified_k__norank_d__Bacteria* sp. These shifts in microbial communities highlight the impact of plastic aging on gut microbiota composition and their functional potential. The enrichment of *Spiroplasma* sp. in the PE1 group and *Lactobacillus* sp. in the PLA1 group suggests that these genera may possess specific metabolic adaptations, which enable them to depolymerize aged plastics more effectively. This observation supports the hypothesis that physical and chemical changes in plastics due to aging, such as surface oxidation and fragmentation, create more favorable conditions for microbial colonization and plastic depolymerization.

### 3.7. Correlations Between Bacterial Genera

**Figure 6:**
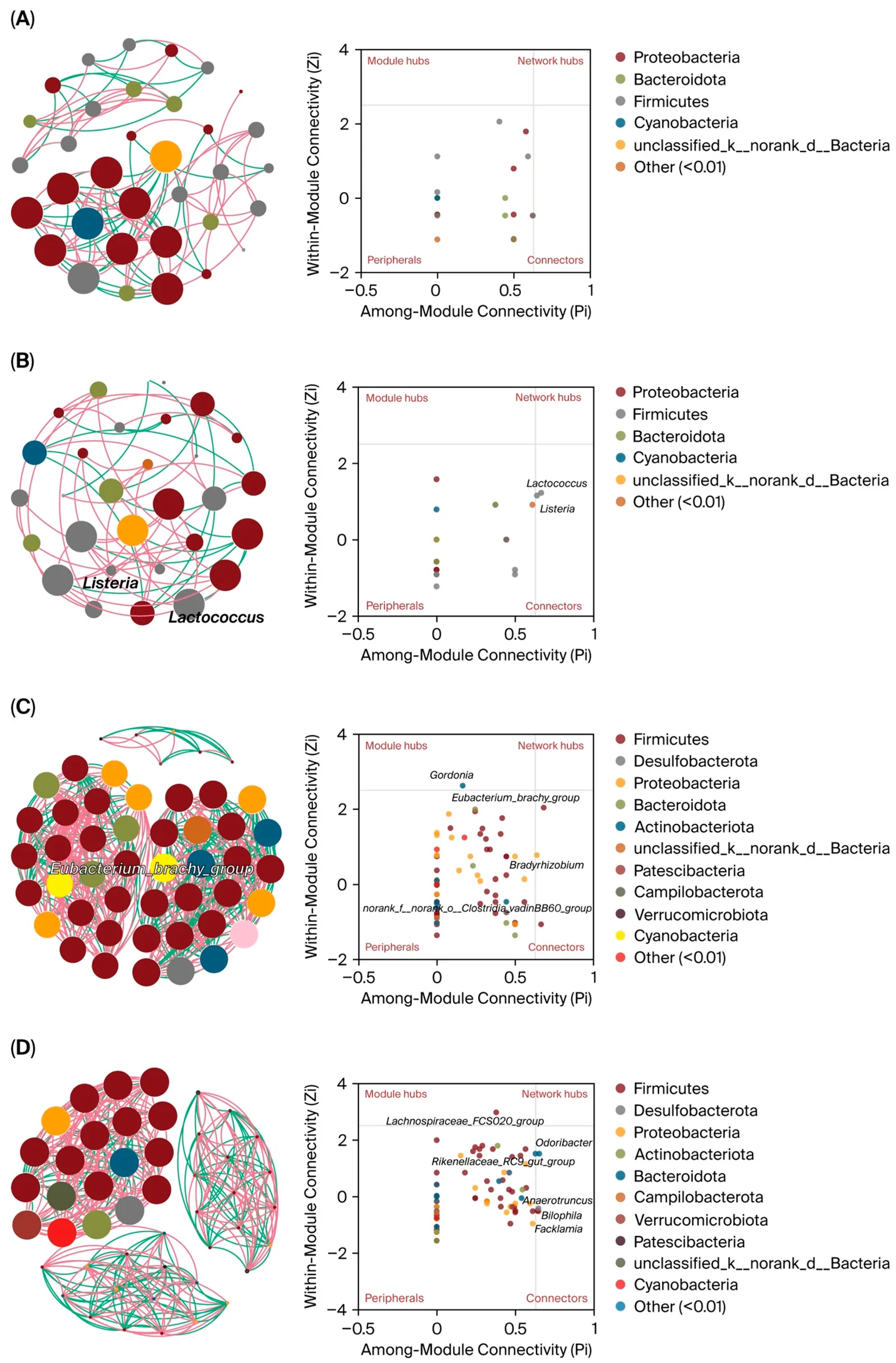
Correlation-based network analysis and ZiPi plot of gut bacterial genera in PEcon (**A**), PE1 (**B**), PLAcon (**C**), and PLA1 (**D**) groups. Node sizes are proportional to the average relative abundance of corresponding taxa in all samples. Red lines represent positive correlations, while green lines represent negative correlations. ZiPi plot displaying the categories of all OTUs according to their topological roles, the size of the circle represents the average relative abundance of the species.

The impact of plastic aging and type on microbial community dynamics was investigated in this study, with a particular focus on interspecies competition and cooperation, which are commonly observed in natural bacterial communities [5,7,54,55,56,57]. Negative and positive interaction networks of microbial genera under different plastic diets were analyzed using Spearman’s correlations and the ZiPi method to identify key taxa. The results demonstrated that specific diets shaped distinct microbial communities, with network analysis at the top 50 genus level revealing varied co-occurrence patterns within the gut microbial communities under different diets. Notably, aging treatment facilitated the emergence of keystone microbial genera (Table S4 and Figure 6).

Microbial networks associated with PE diets showed a simpler and more cooperative pattern overall (Figure 6). The PEcon group (35 nodes, 103 edges) had a more complex network than the PE1 group (30 nodes, 61 edges). Similarly, the PLAcon group displayed a more intricate network (49 nodes, 456 edges) compared to PLA1 (47 nodes, 353 edges) (Table S4). These results indicate the significant influence of specific plastic diets on microbial community interactions, which aligns with the α-diversity patterns observed across the PEcon, PE1, PLAcon, and PLA1 groups (Table S3). Previous studies on the gut microbiota of *Tenebrio* species and *Drosophila melanogaster* involved in the transformation of low-density polyethylene (LDPE) foam and PS MPs have also indicated that co-exclusion effects are linked to increased species diversity, likely driven by heightened competition for limited resources [5,22,57,58,59]. In this study, the PEcon and PLAcon groups displayed more negatively correlated networks than the PE1 and PLA1 groups. This may be attributed to the aging treatment, where freezing followed by UV exposure altered the plastic structure, increasing its bioavailability and reducing competition among gut microbes for essential nutrients and energy (Tables S2 and S4).

Additionally, the PEcon diet showed a more complex co-occurrence pattern with multiple interaction modules (Figure 6A). However, no module hubs, connectors, or network hubs—typically considered keystone taxa—were identified [45]. In contrast, the PE1 diet led to the emergence of *Lactococcus* sp. and *Listeria* sp. as connectors (Figure 6B). In the PLA groups (Figure 6C,D), the gut microbiota exhibited more complex interspecies interactions, especially after aging treatment, suggesting enhanced functional differentiation that may be associated with improved resource utilization efficiency. The PLAcon network contained three connectors (*Eubacterium_brachy_group* sp., *norank_f__norank_o__Clostridia_vadinBB60_group* sp., and *Bradyrhizobium* sp.) and one module hub (*Gordonia* sp.) (Figure 6C). The PLA1 network included five connectors (*Anaerotruncus* sp., *Bilophila* sp., *Odoribacter* sp., *Facklamia* sp., and *Rikenellaceae_RC9_gut_group* sp.) and one module hub (*Lachnospiraceae_FCS020_group* sp.) (Figure 6D). These findings are associated with the observed shifts in microbial community composition under different plastic diets, as indicated by the relative abundance levels of *Spiroplasma* sp. and *Lactobacillus* sp. in PE1 and PLA1 groups, respectively (Figure 4C). However, the functional roles of microbial taxa in plastic degradation cannot be directly inferred solely from abundance data. *Spiroplasma* sp. (>1%) and *Chryseobacterium* sp. (<1%) were synergistic genera in the PEcon group (Supplementary Materials Sheet S1), while *Spiroplasma* sp. (>1%) was the only dominant genus in the PE1 group (Supplementary Materials Sheet S2). No direct functional involvement of *Spiroplasma sp.* in PE degradation can be confirmed based on the current data, and its ecological role remains to be further validated. *Lactobacillus* sp. (>1%)*, Faecalibaculum* sp. (>1%), and *Coriobacteriaceae*_UCG-002 sp. (>1%) were the synergistic genera in the PLAcon group (Supplementary Materials Sheet S3), while *Lactobacillus* sp. (>1%), *Morganella* sp. (<1%), and *Colidextribacter* sp. (>1%) were the synergistic genera in the PLA1 group (Supplementary Materials Sheet S4). These results suggest potential associations between microbial interactions and adaptation to plastic diets. However, the role of specific taxa as plastic-degrading functional strains remains speculative without functional validation (e.g., metagenomics, enzyme assays, or isolation studies).

## 4. Conclusions and Limitations

In this study, we provide valuable insights into the effects of plastic aging and type on the gut microbial community interactions in *T. molitor* larvae, with a focus on the role of interspecies competition, cooperation, and keystone taxa. Our findings demonstrate that plastic aging promoted the ingestion and biodegradation rates of *T. molitor* larvae and increased the PE and PLA removal efficiencies to a limited extent. Although the observed effect sizes were relatively modest, the consistency across multiple independent indicators suggests a reproducible influence of aging-induced physicochemical changes on plastic processing by larvae. Compared with previous studies on PS degradation by *T. molitor* and other insect-mediated plastic biodegradation systems, the present study extends current understanding by demonstrating that different polymer types (PE vs. PLA) exhibit distinct microbiome assembly patterns and network structures under identical aging treatments, rather than a uniform “aging-enhanced degradation” response. Relative to earlier reports showing UV-enhanced PE and PS biodegradation or co-diet-assisted PE transformation, our results indicate that the improvement produced by freezing–UV aging was measurable but limited in terms of consumption and removal rate, while the clearest contribution was the polymer-specific linkage between physicochemical aging, depolymerization indicators, and gut microbial restructuring. In particular, aged PE induced a stronger shift in microbial network simplicity and the dominance of Proteobacteria, whereas aged PLA favored Firmicutes- and Desulfobacterota-associated community restructuring, suggesting polymer-dependent ecological filtering effects rather than a universal microbial response. The results also demonstrated that plastic aging promoted the emergence of key microbial genera, whereas distinct plastic types generated disparate co-occurrence patterns at the microbial network level. The simpler and more cooperative networks in PE groups contrasted with the more complex interactions observed in PLA groups, suggesting that plastic type plays a critical role in shaping microbial communities. Notably, aging treatments altered the bioavailability of plastics and reshaped gut microbial interactions. The main scientific contribution of this work is its integrated comparison of a petroleum-based polymer (PE) and a bio-based polymer (PLA) under the same accelerated aging and larval-feeding framework, linking polymer physicochemical shifts, partial depolymerization, and deterministic gut microbiome assembly into a single mechanistic assessment. This study also establishes a polymer-specific framework that links aging-induced physicochemical modifications, depolymerization behavior, and gut microbiome assembly under identical PE and PLA processing conditions. Our scientific contribution is not that freezing–UV aging alone provides a practical treatment solution, but that identical aging and larval-feeding conditions reveal different PE and PLA pathways connecting surface chemistry, polymer chain changes, and deterministic gut microbiome restructuring.

Despite these significant findings, this study has several limitations. First, a limitation of this study is the absence of co-feeding treatments combining plastics with nutrient substrates such as WB. Such co-feeding systems have alleviated nutritional stress in previous studies and may enhance larval growth and plastic processing efficiency. Therefore, the present results should be interpreted as the effects of plastic type and aging under simplified dietary conditions, rather than optimized degradation performance under nutrient-supplemented systems. The experimental design focused primarily on two types of plastics (PE and PLA), which limits the generalizability of the present results to other plastic types. Second, the effects were examined only under specific aging conditions (freezing and UV exposure), which may not fully represent natural environmental processes such as rainfall, humidity changes, snow-cover shielding, soil exposure, mechanical abrasion, and day–night or seasonal temperature fluctuations. Finally, although aging promoted ingestion and increased plastic processing rates by *T. molitor* larvae, its effect on overall polymer transformation remained limited. Future studies should incorporate co-feeding designs to further elucidate the synergistic effects of dietary nutrients on plastic degradation. Furthermore, it is important to explore a broader range of plastic types and environmental aging conditions, as well as to investigate potential synergistic interactions between plastic-degrading microbes and other microbial taxa. Future research integrating isotope tracing, CO_2_ evolution measurements, and targeted metabolite analyses will be necessary to quantify plastic mineralization and elucidate degradation pathways. Additionally, developing strategies to enhance microbial cooperation may further improve plastic degradation efficiency, offering promising avenues for mitigating the environmental impact of plastic waste. Future work should test naturally weathered plastics collected from field environments and compare them with controlled aging treatments to determine how rainfall, snow-cover shielding, soil exposure, humidity, mechanical abrasion, and seasonal temperature fluctuations modify larval processing and gut microbiome responses.

## Data Availability

The raw data supporting the conclusions of this article will be made available by the authors on request.

## Supplementary Materials

The following supporting information can be downloaded at: https://www.mdpi.com/article/10.3390/microorganisms14081700/s1, Method S1: Calculation of weight change, final SR, and SPCR; Method S2: Procedures of frass collection and measurement of PS particles; Method S3: Detailed GPC analysis; Method S4: Detailed FTIR analysis; Method S5: Detailed TGA analysis; Method S6: Detailed operation procedure for gut obtains, DNA extraction, and PCR amplification in gut microbiome analysis; Method S7: Calculation of solar simulation equivalent; Figure S1: Mealworms and plastics; Table S1: Summary of molecular weight (kDa) of PEcon, PLAcon, PE1, and PLA1 MPs used for biodegradation test; Table S2: Summary of properties of aged MPs and then consumption and depolymerization by mealworms; Table S3: Bacterial diversity analyses based on illumine sequencing of the 16S rRNA gene amplicons by all tested larvae; Table S4: Bacterial correlation analyses based on illumine sequencing of the 16S rRNA gene amplicons by top 50 genera of mealworms; topological roles of gut bacterial genus according to ZiPi plot in PEcon (Sheet S1), PE1 (Sheet S2), PLAcon (Sheet S3), and PLA1 (Sheet S4) groups.

## Abbreviations

The following abbreviations are used in this manuscript:

PE	Polyethylene
PLA	Polylactic acid
MPs	Microplastics
PS	Polystyrene
NPs	Nanoplastics
MW	Molecular weight
WB	Wheat bran
M_n_	Number-average molecular weight
M_w_	Weight-average molecular weight
OTUs	Operational taxonomic units
PCoA	Principal coordinate analysis
NST	Normalized Stochasticity Ratio
db-RDA	Distance-based redundancy analysis
VPA	Variation partitioning analysis
