# Supplementary material for: Aging-Induced Physicochemical Changes in Petroleum- and Biobased Microplastics Influence Depolymerization and Gut Microbiota in Tenebrio molitor Larvae
Source: Microorganisms. 2026 Aug 3;14(8):1700. doi: 10.3390/microorganisms14081700 (PMC13515386; doi:10.3390/microorganisms14081700)
Supplement: Supplementary file 1 [file microorganisms-14-01700-s001.zip › Revised SI.pdf]

*Supporting Information*

# Aging-Induced Physicochemical Changes in Petroleum- and Biobased Microplastics Influence Depolymerization and Gut Microbiota in *Tenebrio molitor* Larvae

**Yuan Tian [1,2], Meng-Qi Ding [1,2,3,*], Jie Ding [1,2], Xin-Ran Ren [2], Sheng-Qiang Fan [1,2], Bing-Feng Liu [1,2], De-Feng Xing [1,2], Lei Zhao [1,2], Zhi-Rong Zhang [4], Lu-Yan Zhang [5], Nan-Qi Ren [1,2] and Shan-Shan Yang [1,2,*]**

[1] National Engineering Research Center for Safe Disposal and Resources Recovery of Sludge, Harbin Institute of Technology, Harbin 150090, China; raintian1990@foxmail.com (Y.T.); dingjie123@hit.edu.cn (J.D.); fsq@hit.edu.cn (S.-Q.F.); lbf@hit.edu.cn (B.-F.L.); dxing@aliyun.com (D.-F.X.); lzhao@hit.edu.cn (L.Z.); rnq@hit.edu.cn (N.-Q.R.)

[2] State Key Laboratory of Urban-rural Water Resource and Environment, Harbin Institute of Technology, Harbin 150090, China; 21s029004@stu.hit.edu.cn

[3] State Key Laboratory of Water Engineering Ecology and Environment in Arid Area, College of Water Conservancy and Civil Engineering, Inner Mongolia Agricultural University, Hohhot 010018, China

[4] College of Forestry, Inner Mongolia Agricultural University, Hohhot 010018, China; zhang_zr@126.com

[5] School of Environmental Science and Engineering, Yancheng Institute of Technology, Yancheng 224051, China; zly3161@163.com

* Correspondence: mengqiding@imau.edu.cn (M.-Q.D.); shanshanyang@hit.edu.cn (S.-S.Y.)

**Methods (sample treatment and instrumental conditions)**

**Method S1. Calculation of weight change, final SR and SPCR**

The larval average weight change, survival rate (SR) and specific plastic consumption rate (SPCR) were counted after 24 days of incubation.

Larval average weight change = $(W_f - W_i)/ W_i \times 100\%$ (1)

where $W_i$ is the larval average initial weight and $W_f$ is the larval average final weight.

SR was calculated as follows:

SR (%) = Ns/N × 100% (2)

where N is the initial number of mealworms (150 mealworms in each incubator); $N_S$ is the number of live mealworms (larvae plus pupae and live beetles if any) on day 24.

SPCR was calculated on the basis of the mass of plastic consumed over the test period (24 days) and the initial number of larvae.

SPCR (mg plastic/100 larvae per day) = $W_{plastic} \times 100/(150 \times 24)$ (3)

where SPCR is the specific plastic consumption rate (mg plastic/100 larvae per day); $W_{plastic}$ is the total mass of Plastic consumed.

- **Method S2. Procedures of frass collection and measurement of PS particles**

Frass was collected by relocating the larvae to clean containers after cleaning their respective containers with compressed air to eliminate residual feeds. The frass was weighed after drying and stored at -20°C in a refrigerator (BCD-215WTM, Media, Shenzhen, China) for further analysis.

The procedure involved collecting approximately 1g of frass into a 100ml glass vial, followed by the addition of 30 ml of 30% hydrogen peroxide solution and

incubation in a constant temperature chamber at 60°C for 24 hours to degrade organic matter. The resulting solid mixture was transferred to a clean 100ml glass vial. Subsequently, 30ml of 1.2 mol/L NaCl solution was added for thorough density separation of plastics. This process was repeated three times to obtain PE/PLA microplastics from the frass(Glaser, 2019).

The residual plastic in frass ($W_{plastic\text{-}frass}$, mg) was then calculated on the basis of increase in the weight of the filter (g). The content of plastic in frass ($C_{plastic}$, %, w/w) is calculated based on the residual plastic extracted (mg) versus in dry weight of frass sample.

$$C_{PS}\ (\%, w/w) = W_{plastic\text{-}frass}/W_{frass} \times 100\ \%\ (4)$$

**Method S3. Detailed of GPC analysis**

To characterize depolymerized polymer in the frass, we determined the molecular weights ($M_n$ and $M_w$) using gel permeation chromatography (GPC) (PL GPC 50, Agilent Technologies, Inc., USA). After drying at ambient temperature, 100 mg of Frass and PLA samples were gently crushed in a mortar and pestle before a 4-hour extraction in 4 mL of chloroform solvent (HPLC grade purity ≥99.9%, Merck KGaA, Darmstadt, Germany). After a 4-hour extraction at 50°C, the solvent was filtered through a stainless-steel filter and transferred into a clean glass vial. The polymer was then dried, weighed, and re-dissolved in chloroform solvent to achieve a final concentration of 1.2 mg/mL before being injected into GPC. Triplicate analyses were performed at 40°C using a 100 μL injection volume with a chloroform flow rate of 1.0 mL/min.

HT-GPC (HT-GPC, PL GPC 220, Agilent Technologies, Inc., USA) was applied to

analyze changes in the molecular weight of the PE. 100 mg of Frass and PE samples was dissolved in 1,2,4-TCB (≥ 99%) to obtain a final concentration of approximately 1.2 mg/ml. Each sample was analyzed at 150 °C using a 200 μl injection volume with an eluent (1,2,4-TCB) flow rate of 1.0 ml/min. Triplicate HT-GPC analyses were carried out to determine $M_w$, $M_n$, and molecular weight distribution (MWD) of feedstock and frass samples.

The reduction of molecular weight (%) was calculated as:

$$R\ (\%) = (M_o - M_f)/M_o \times 100\% \quad (5)$$

where R is the change (%), $M_o$ is molecular weight of the virgin PLA/PE sample, and $M_f$ is the molecular weight of residual PLA/PE extracted from frass. When molecular weight decreases, *R* is expressed as a positive value. When molecular weight increases, the *R* value becomes negative.

**Method S4. Detailed of FTIR analysis**

Fourier Transform Infrared Spectroscopy (FTIR) analysis (Nicolet iS50 FTIR Spectrometer, Thermo Fisher Scientific, U.S.A.) was used to characterize the major functional groups in PS feedstocks and the corresponding residual polymers from frass samples. Spectra were recorded from the residual polymers extracted from the frass ground to a homogeneous powder in absorbance mode and transformed into transmittance for graphing. Spectra were recorded in the range of 400-4,000 $cm^{-1}$ with a minimum of 16 scans with a spectral resolution of 0.6329 $cm^{-1}$. Peaks were identified using OMNIC software (Thermo Fisher Scientific Inc., Pittsburgh, PA).

**Method S5. Detailed TGA analysis**

Biodegradation of PE and PLA is indicated by a decrease in stability. Plastic feedstock and frass samples containing residual PE/PLA were analyzed by TGA (TG-DSC, NETZSCH STA 449 F3, Germany). Samples were heated from 40 to 800 °C at a rate of 10 K/min under a high-purity nitrogen ambience (99.999%).

**Method S6. Detailed operation procedure for gut obtains, DNA extraction and PCR amplification in Gut microbiome analysis.**

At the end of the 24-day test, 30 larvae were randomly selected from each treatment for gut microbial community analysis. Prior to gut dissection, larvae were starved for 24 h to allow clearance of transient gut contents and reduce contamination from residual diet particles. The larvae were decontaminated by immersing them in 75% ethanol for 1 minute, then rinsed twice with 0.01M phosphate buffer saline (PBS) or sterilization. All dissections were conducted under a laminar flow biosafety cabinet using sterilized instruments to minimize environmental contamination. The gut tissue was dissected and harvested, then placed in a 2 mL centrifuge tube and homogenized. All samples were stored in a -80 ℃ refrigerator (Revco PLUS, Thermo Fisher Scientific, Waltham, US) for further analysis. Total bacterial genomic DNA samples were extracted from larval gut using the Fast DNA SPIN extraction kits (Illumina, San Diego, CA, USA) after homogenization of intestinal samples following the manufacturer's instructions. Each group was divided into four samples for testing and stored at −20°C for further analysis. The quantity and quality of extracted DNA were measured using a NanoDrop ND-1000 spectrophotometer (Thermo Fisher Scientific, Waltham, MA, USA) and

agarose gel electrophoresis, respectively. The PCR amplification of the bacterial 16S rRNA gene V3–V4 region was performed using the forward primer 338F (5'-ACTCCTACGGGAGGCAGCA-3') and the reverse primer 806R (5'-GGACTACHVGGGTWTCTAAT-3'). Sample-specific 7-bp barcodes were incorporated into the primers for multiplex sequencing. The PCR components contained 5 μl of Q5 reaction buffer (5×), 5 μl of Q5 High-Fidelity GC buffer (5×), 0.25 μl of Q5 High-Fidelity DNA Polymerase (5U/μl), 2 μl (2.5 mM) of dNTPs, 1 μl of each Forward and Reverse primer (10 uM), 2 μl of DNA Template, and 8.75 μl of $ddH_2O$. Thermal cycling consisted of initial denaturation at 98 °C for 2 min, followed by 25 cycles of denaturation at 98 °C for 15 s, annealing at 55 °C for 30 s, and extension at 72 °C for 30 s, with a final extension of 5 min at 72 °C. Thereafter, PCR amplicons were purified with the Agencourt AMPure Beads (Beckman Coulter, Indianapolis, IN) and purified amplicons were paired-end sequenced on an Illumina MiSeq platform (Illumina, San Diego, CA, USA). After the individual quantification step, amplicons were pooled in equal amounts, and pair-end 2×300 bp sequencing was performed using the Illumina MiSeq platform with MiSeq Reagent Kit v3 using the free online Majorbio Cloud Platform.

**Method S7. Calculation of solar simulation equivalent**

Intensity (here UVC) [W/m$^2$] × tims of exposure [s] = total irradiance exposed [J/m$^2$]

$$\text{Harbin winter mean irradiance} \approx 5982\ \text{kJ/(m}^2 \times \text{d)}$$

$$5\ \%\ \text{of this is UV light: Harbin winter UV irradiance} = 29.91\ \text{J/(cm}^2 \times \text{d)}$$

The total light intensity was set at 2493 μW/cm$^2$ by UV Meter (UVC254E,

XINBAOKEYI, China).

**Formula:** total irradiance exposed / mean Harbin winter UV irradiance = simulated days (6)

2493 μW/$cm^2$ (value at a distance of 10 cm) × (10 × 10 × 60 × 60) s = 897.48 J/$cm^2$ (UV irradiance value)

897.48 (J/$cm^2$)/ 29.91 J/($cm^2$ × d) ≈ 30 days simulated during 10 days.

Harbin winter irradiance in 2023 from http://www.maps4gis.com

| Month | November | December | January | Average |
|---|---|---|---|---|
| **Intensity, kJ/($m^2$ × d)** | 6816 | 5069 | 6060 | 5982 |

**Figure**

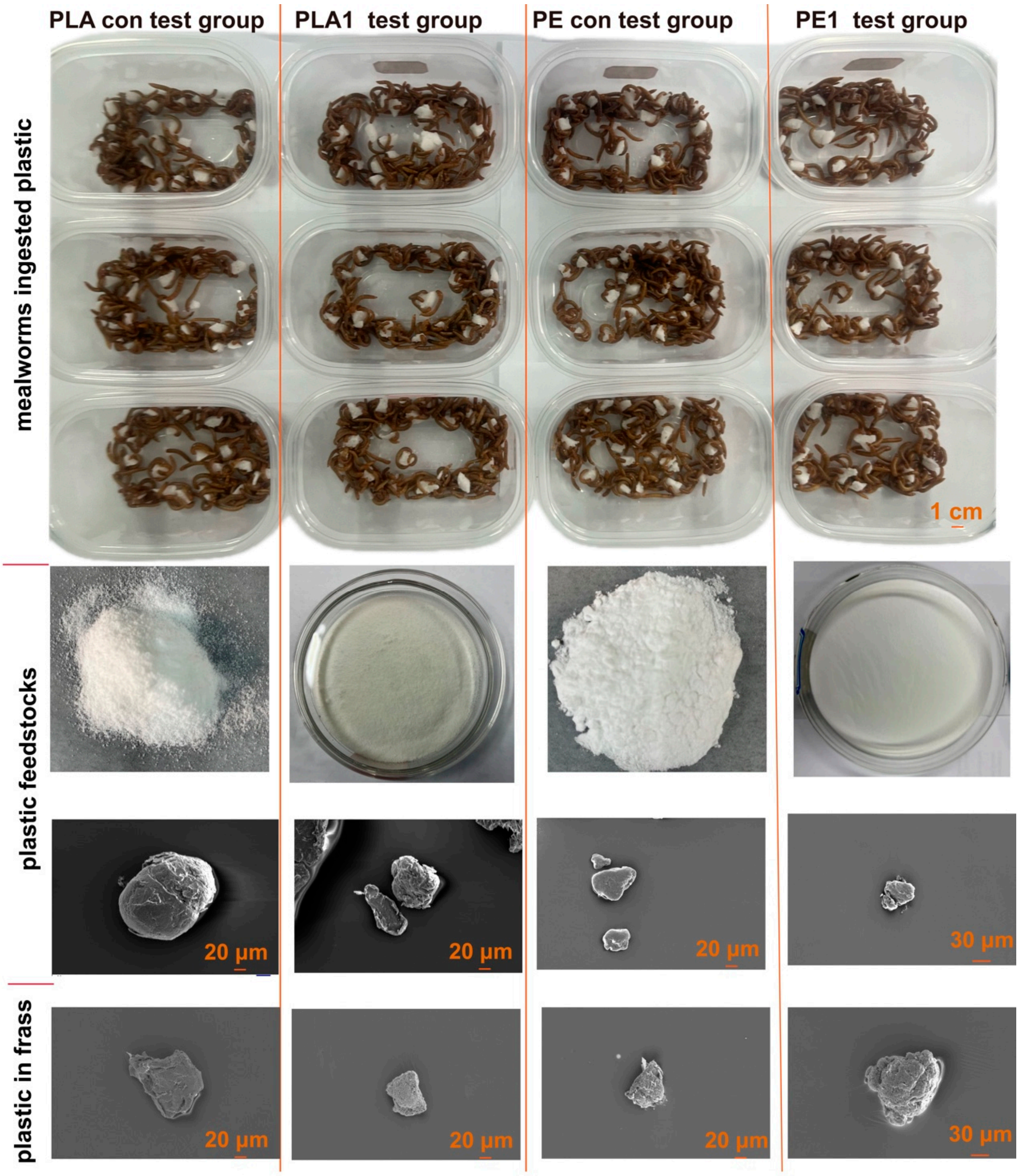


**Figure S1.** Mealworms and plastics.

## Tables

**Table S1.** Summary of molecular weight (kDa) of PEcon, PLAcon, PE1 and PLA1 MPs used for biodegradation test.

| | PE con | PE1 | PLA con | PLA1 |
|---|---|---|---|---|
| $M_w$ | 124.4 ± 0.5 | 124.0 ± 0.2 | 186.6±2.2 | 183.4±4.3 |
| $M_n$ | 8.6 ± 0.6 | 8.5 ± 0.2 | 132.7±1.2 | 126.9±2.0 |

**Table S2**. Summary of properties of aged MPs and then consumption and depolymerization by mealworms.

| | Initial larval average weight, mg | Final larval average weight, mg | Survival rate, % | PS consumption, g | SPCR | Removal rates, % | $M_n$ of frass, kDa | $M_w$ of frass, kDa |
|---|---|---|---|---|---|---|---|---|
| PEcon | 61 ±1 | 54 ±1 | 74 ±1.03 | 20.9 ±0.5 | 829.6 ±15.8 | 53.8 ±2.4 | 7.8 ±0.7 | 121.2 ±1.7 |
| PE1 | 63 ±3 | 60 ±1 | 80 ±1.37 | 23.2 ±0.35 | 859.3 ±7.6 | 55.6 ±5.0 | 7.8 ±0.9 | 101.6 ±1.8 |
| PLAcon | 62 ±1 | 74 ±1 | 70±1.27 | 19.1 ±0.9 | 717.1 ±12.9 | 80.8 ±3.9 | 62.2 ±1.9 | 143.1 ±4.3 |
| PLA1 | 61 ±2 | 78 ±1 | 75±0.64 | 21.1 ±0.8 | 732.8 ±12.2 | 81.6 ±0.4 | 59.9 ±1.7 | 142.7 ±1.1 |
| WB | 61 ±1 | 80 ±1 | 86±1.4 | nd | nd | nd | nd | nd |

[#]The initial larval number was 150. Specific plastic consumption was calculated on the basis of the mass of plastic consumed over the test period (24 days) and the initial number of larvae; nd = not determined

**Table S3.** Bacterial diversity analyses based on illumine sequencing of the 16S rRNA gene amplicons by all tested larvae.

| Estimators | PE1 | PEcon | PLA1 | PLAcon | WB |
|---|---|---|---|---|---|
| Ace | 44 ±8.8 | 45.4 ±9.3 | 181.4 ±12.4 | 179.1 ±27.9 | 150.8 ±95.6 |
| Chao | 41.5 ±7.1 | 41.9 ±3.8 | 177.7 ±13.2 | 176.7 ±31.4 | 145.3 ±95.8 |
| Coverage | 1 ±0 | 1 ±0 | 1 ±0 | 1 ±0 | 1 ±0 |
| Shannon | 1.6 ±0.3 | 2 ±0.1 | 2.5 ±0.6 | 2.4 ±0.4 | 1.5 ±0.6 |
| Simpson | 0.3 ±0.1 | 0.2 ±0 | 0.2 ±0.2 | 0.2 ±0.1 | 0.4 ±0.2 |
| Sobs | 32.3 ±3.5 | 37.7 ±4 | 156.7 ±14.6 | 158.3 ±30.2 | 133 ±93 |

**Table S4.** Bacterial Correlation analyses based on illumine sequencing of the 16S rRNA Gene amplicons by mealworms top 50 genera.

| Samples | Node Number | Edge Number | Positive Coefficient (%) | Negative Coefficient (%) |
|---|---|---|---|---|
| PEcon | 35 | 103 | 57.3 | 42.7 |
| PE1 | 30 | 61 | 67.2 | 32.8 |
| PLAcon | 49 | 456 | 54.4 | 45.6 |
| PLA1 | 47 | 353 | 56.7 | 43.3 |
